# Avaliação do Tempo de Condução Atrioventricular Dinâmica para Acoplamento ao Intervalo RR em Atletas e Indivíduos Sedentários

**DOI:** 10.36660/abc.20190281

**Published:** 2020-07-28

**Authors:** Paulo Roberto Benchimol-Barbosa, Olivassé Nasario, Jurandir Nadal

**Affiliations:** 1 Hospital Universitário Pedro Ernesto Rio de JaneiroRJ Brasil Hospital Universitário Pedro Ernesto, Coordenação Clínica, Rio de Janeiro, RJ – Brasil; 2 Universidade Federal do Rio de Janeiro Instituto Alberto Luiz Coimbra de Pós-Graduação e Pesquisa de Engenharia Rio de JaneiroRJ Brasil Universidade Federal do Rio de Janeiro, Instituto Alberto Luiz Coimbra de Pós-Graduação e Pesquisa de Engenharia, Rio de Janeiro, RJ – Brasil

**Keywords:** Atletas, Adultos, Treinamento de Resistência, Aptidão Física, Remodelação Ventricular, Sedentarismo, Eletrocardiografia/métodos, Ventrículos do Coração, Função Ventricular

## Abstract

**Fundamento:**

O tempo de condução atrioventricular (TCAV) é influenciado pelo estímulo autonômico e sujeito a remodelação fisiológica.

**Objetivo:**

Avaliar a variabilidade da TCAV batimento-a-batimento e o intervalo RR em atletas e indivíduos sedentários saudáveis.

**Métodos:**

Vinte adultos, incluindo 10 indivíduos sedentários saudáveis (controles) e 10 corredores de elite de longa distância (atletas), com idade, peso e altura ajustados foram submetidos à avaliação do equivalente metabólico máximo (MET) e registro de ECG em repouso supino de 15 minutos sete dias depois. O intervalo entre os picos da onda P e da onda R definiu o TCAV. Foram calculadas a média (M) e o desvio padrão (DP) de intervalos RR consecutivos (RR) e TCAV acoplados, bem como as linhas de regressão de RR vs. TCAV (RR-TCAV). A condução AV concordante foi definida como o *slope* RR-AVCT positivo e, caso contrário, discordante. Um modelo de regressão linear multivariada foi desenvolvido para explicar o MET com base nos parâmetros de variabilidade do TCAV e intervalo RR. Nível de significância: 5%.

**Resultados:**

Nos atletas, os valores de M-RR e DP-RR foram maiores que nos controles, enquanto M-TCAV e DP-TCAV não foram. Os *slopes* RR-TCAV foram, respectivamente, 0,038 ± 0,022 e 0,0034 ± 0,017 (p < 0,05). Utilizando um valor de corte de 0,0044 (AUC 0,92 ± 0,07; p < 0,001), o *slope* RR-TCAV mostrou 100% de especificidade e 80% de sensibilidade. Em um modelo multivariado, o *slope* DP-RR e RR-TCAV foram variáveis explicativas independentes do MET (razão F: 17,2; p < 0,001), apresentando especificidade de 100% e sensibilidade de 90% (AUC: 0,99 ± 0,02; p < 0,001).

**Conclusão:**

Em corredores de elite, o acoplamento dinâmico de TCAV para intervalo RR apresenta condução AV discordante espontânea, caracterizada por *slope* na linha de regressão TCAV negativa vs. intervalo RR. O desvio padrão dos intervalos RR e o *slope* da linha de regressão do TCAV vs. intervalo RR são variáveis explicativas independentes do MET. (Arq Bras Cardiol. 2020; [online].ahead print, PP.0-0)

## Introdução

A adaptação cardíaca secundária à aptidão física se reflete na remodelação mecânica, elétrica e autonômica do coração, como consequência de repetidas atividades de alta demanda. Atletas bem treinados costumam apresentar discreto ganho de massa ventricular, aumento da amplitude da onda do ECG, repolarização precoce, redução da frequência cardíaca em repouso e aumento da variabilidade da frequência cardíaca, relacionados ao status de condicionamento.^1 - 7^

Particularmente, a maior parte do remodelamento autonômico do coração em atletas bem condicionados é consequência do aumento do tônus vagal e da estimulação simpática reduzida sobre o seio e os nós atrioventriculares (AV).^1 , 6^ Embora o aumento do tônus vagal possa ser detectado diretamente pela medida da frequência cardíaca em repouso, diferenciar entre o aumento da atividade parassimpática sobre o nó sinusal e o nó AV no ECG de superfície pode não ser tão simples.

Frequentemente, atletas de alta demanda apresentam remodelamento do nó atrioventricular (AV), caracterizado por vários graus de bloqueio de condução AV, ritmo atrial ou juncional baixo não-sinusal e, mais raramente, bloqueio AV completo.^8 - 10^ Esses distúrbios da condução AV dependem do status do condicionamento físico e estão relacionados não apenas ao aumento da atividade parassimpática sobre o nó AV, mas também ao remodelamento secundário das fibras do nó AV e ao acoplamento célula a célula.^11 - 13^

Embora os distúrbios da condução AV tenham sido documentados repetidamente em atletas, a adaptação dinâmica da condução AV ao ciclo cardíaco nessa população ainda precisa de esclarecimentos. Na população geral, a duração AV varia dinamicamente de acordo com a duração do intervalo RR, caracterizando um efeito semelhante a uma sanfona. Entretanto, em atletas, o remodelamento autonômico pode influenciar a condução dinâmica AV na adaptação do intervalo RR, levando a um comportamento distinto da condução AV, em uma resposta à variação do intervalo RR relacionada ao tempo.

O presente estudo avaliou as variabilidades do tempo de condução AV (TCAV) batimento por batimento e do intervalo RR em corredores de elite e indivíduos sedentários saudáveis, em repouso, com o objetivo de avaliar o efeito do status da aptidão física na duração do acoplamento espontâneo de TCAV ao intervalo RR.

## Métodos

Informações detalhadas sobre o protocolo do estudo, aprovação do Comitê de Ética e aquisição de dados do ECG foram descritas em outro artigo.^6^ O presente estudo analisou dados brutos de ECG de alta resolução do banco de dados de ECG do Programa de Engenharia Biomédica, utilizando uma nova técnica para extração do tempo de condução atrioventricular e intervalos RR.^14^ O procedimento de amostragem de dados foi descrito em outro artigo.^6^

A população do estudo foi composta por 20 voluntários divididos em dois grupos: ‘Atleta’, composto por dez corredores de elite de longa distância (≥ 16,0 equivalentes metabólicos máximos [MET]) calculados como o consumo máximo de oxigênio obtido durante o teste de estresse dividido por 3,5 mL·kg^-1^·min^-1^, [média ± DP] 19,5 ± 1,3 MET; idade 25,1 ± 7,1 anos) e ‘Controle’, constituído por dez voluntários sedentários saudáveis do sexo masculino (≤ 11,5 MET; 8,7 ± 1,9 MET; idade 29,0 ± 5,4 anos). Vale ressaltar que o termo ‘MET’ é empregado ao longo do texto como o equivalente metabólico máximo alcançado durante o teste de estresse. O programa de treinamento dos atletas consistiu em seis a oito sessões de treinamento / semana; 90 a 120 min / sessão; 90 a 110 km / semana A detecção das ondas e do ponto fiducial foi realizada no ECG realizado com derivações XYZ de Frank modificadas em posição supina em repouso, usando filtro passa-baixa a 15 Hz (Butterworth, 4ª ordem). Para a análise da duração do intervalo RR, artefatos e batimentos ectópicos foram adequadamente excluídos.^15 , 16^

A distância entre o pico da onda P e o pico da onda R em batimentos normais definiu o intervalo PR-pico e foi utilizada para analisar a adaptação instantânea do TCAV ao longo do ciclo cardíaco.^14^ O acoplamento PR-pico a intervalos RR foi avaliado batimento a batimento durante todo o registro do ECG, utilizando a derivação que mostra a onda P mais alta, geralmente a derivação Y. O intervalo RR foi avaliado como o tempo entre os picos das ondas R de dois batimentos normais consecutivos. O intervalo PR-pico foi avaliado imediatamente antes do segundo batimento do respectivo intervalo RR. Para cada indivíduo, foi calculada a média (M) e o desvio padrão (DP) de todos os intervalos RR normais consecutivos (M-RR e DP-RR) e o respectivo intervalo PR-pico (M-TCAV e DP-TCAV). Os intervalos PR-pico foram correlacionados com os respectivos intervalos RR e o slope (inclinação) das linhas de regressão foi calculado (RR-TCAV_slope_).

### Análise Estatística

As variáveis foram expressas como média ± DP ou mediana e variação interquartil, quando apropriado. A normalidade dos dados foi avaliada pelo teste de Kolmogorov-Smirnov, e todas as variáveis analisadas tiveram sua premissa de normalidade aceita. As variáveis foram comparadas entre os grupos usando o teste *t* de Student não pareado. Para avaliar os melhores valores de corte, as curvas ROC foram calculadas a partir do *slope* das linhas de regressão (TCAV vs. intervalo RR) em ambos os grupos. Um modelo de regressão linear multivariada foi desenvolvido para explicar o MET com base em parâmetros significativos de TCAV e intervalo RR. O coeficiente de correlação de Pearson r foi testado quanto à significância (o nível de significância adotado foi de 5%). Uma condução AV concordante foi arbitrariamente definida como TCAV e o intervalo RR aumentou e diminuiu na mesma direção em ciclos cardíacos consecutivos e, de outro modo, foi discordante. O TCAV foi avaliado como intervalo de PR pico.

Um procedimento de reamostragem de validação de *bootstrap* aplicado ao modelo multivariado foi realizado em duas abordagens diferentes. Na primeira abordagem, 1.100 repetições com substituição foram realizadas em toda a amostra de ambos os grupos para avaliar estimativas médias e DP de variáveis independentes. Em uma segunda abordagem, os dois grupos foram divididos em um grupo de teste, composto por 67% dos indivíduos de cada grupo, e um grupo de validação, com os 33% restantes. O MET estimado pelo modelo multivariado foi utilizado para classificar Controles e Atletas em todos os conjuntos de procedimentos de *bootstrap* . Os sinais brutos de ECG foram processados utilizando programas personalizados, escritos na linguagem Matlab R2007a (The MathWorks, Inc). A análise estatística foi realizada utilizando o MS-Excel 360 (Microsoft Corporation) e Medcalc versão 11 (Medcalc Software bvba). O nível de significância adotado na análise estatística foi de 5%.

## Resultados

**Figura 1:**
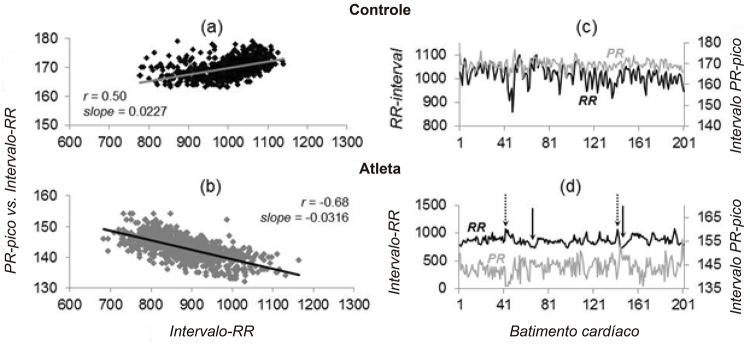
– *Gráfico de dispersão e linha de regressão do intervalo RR batimento a batimento em função do respectivo intervalo PR-pico de um indivíduo Controle de 30 anos (a) e um Atleta de 19 anos de idade (b). Duzentas sequências de batimentos cardíacos das respectivas séries de intervalos de pico RR e PR são mostradas em (c) e (d). Em (a), o intervalo PR-pico aumenta à medida que o intervalo RR aumenta (slope positivo: 0,0227; r = 0,50; p < 0,01), claramente observado em (c) (condição concordante espontânea). Por outro lado, em (b), o intervalo PR-pico diminui à medida que o intervalo RR aumenta (slope negativo: -0,0316; r = -0,68; p < 0,01). Em (d), observe períodos de variação recíproca nos intervalos de pico RR e PR (condução discordante espontânea): o intervalo de pico PR diminui à medida que o intervalo RR aumenta (seta pontilhada) e o intervalo PR-pico aumenta à medida que o intervalo RR diminui (condução decremental, seta sólida) (consulte o texto para mais detalhes).*

Os atletas apresentaram valores de M-RR e DP-RR significativamente maiores que os controles, embora sem diferenças significativas entre os valores de M-TCAV e DP-TCAV ( Tabela 1 ). Exemplos de indivíduos dos grupos Controle (a) e Atletas (b) são apresentados na Figura 1 , onde são apresentadas as linhas de regressão e os respectivos gráficos de dispersão r de TCAV vs. de intervalos RR. O RR-TCAV_slope_ é positivo nos Controles ( Figura 1-a ) e negativo nos Atletas ( Figura 1-b ). No geral, o *slope* RR-TCAV nos Controles e Atletas resultou em diferenças significativas entre os grupos ( Tabela 1 ).

**Tabela 1:** – Análises univariadas das variáveis estudadas (média ± DP)

	*M-RR*	*DP-RR*	*M-TCAV*	*DP-TCAV*	*RR-TCAV_slope_*
**Grupo Controle (média ± DP)**	853,9 ± 94,0	44,5 ± 10,1	134,0 ± 17,7	2,8 ± 1,1	0,0376 ± 0,0219
**Grupo Atleta (média ± DP)**	1079,1 ± 207,9	76,4 ± 21,0	143,3 ± 27,6	3,8 ± 2,2	-0,0034 ± 0,0172
**Nível de Significância p**	0,0084	0,0008	0,3820	0,2032	0,002
**Estatísticas ROC**					
Área sob a curva ROC (AUC)	0,89	0,9	0,56	0,61	0,92
Erro Padrão	0,07	0,08	0,14	0,14	0,06
Intervalo de Confiança de 95%	0,67–0,98	0,68–0,98	0,32–0,78	0,37–0,82	0,71–0,99
Estatística z	5,5	5,2	0,4	0,8	6,7
Nível de Significância p (área = 0.5)	< 0,0001	< 0,0001	0,6721	0,4177	< 0,0001
Valor de corte	917,3	60,9	124,1	3,8	0,0044
Especificidade	80%	100%	40%	50%	100%
Sensibilidade	80%	80%	90%	80%	80%
Exatidão	80%	90%	65%	65%	90%

*M-RR: média de todos os intervalos RR normais; DP-RR: desvio-padrão de todos os intervalos RR normais; M-TCAV: média dos intervalos de PR-pico correspondentes aos intervalos RR normais; DP-TCAV: desvio padrão dos intervalos PR-pico respectivos aos intervalos RR normais; RR-TCAV_slope_: slope da linha de regressão linear entre os intervalos de PR-pico e o respectivo intervalo RR*

As variáveis que apresentaram diferenças significativas entre os grupos foram inseridas em um modelo de regressão linear multivariada, considerando o MET como variável dependente no procedimento bootstrap. DP-RR (p = 0,0099) e RR-TCAV_slope_ (p = 0,006) foram variáveis explicativas independentes do MET, mostrando especificidade de 90%, sensibilidade de 100% e exatidão total de 95% ( Tabela 2 ). A estatística-C média nos grupos de teste e validação foi, respectivamente, 0,97 ± 0,06 e 0,87 ± 0,13; p <0,001 para ambos. A análise de regressão linear multivariada e os respectivos resultados dos procedimentos de *bootstrap* estão resumidos na Tabela 2 .

**Tabela 2:** – Modelo explicativo multivariável do consumo máximo de VO2; 1.100 resultados do procedimento de reamostragem de *bootstrap* e validação interna do modelo explicativo multivariável de consumo máximo de VO2 usando o ‘Teste’ *bootstrap* 2: 1 vs. resultados do procedimento ‘Validação’

	Modelo multivariado		Procedimento de bootstrap		Teste de bootstrap -validação	

Variáveis do Modelo	Coeficientes ±	*p*	Coeficientes ±	*p*	Coeficientes ±	*p*
***Slope* PR a RR (15 Hz)**	-100,36 ± 31,97	0,006	-105,76 ± 33,93	0,0009	-101,42 ± 29,39	0,0003
**DP-RR**	0,115 ± 0,040	0,0099	0,115 ± 0,041	0,003	0,115 ± 0,036	0,0007
**Constante**	8,88		8,75 ± 3,22	0,003	8,85 ± 2,83	0,0009
Estatísticas ROC					Grupo Teste	Grupo Validaçaõ
Área sob a curva ROC (AUC)	0,99		0,99		0,97	0,87
Erro Padrão	0,02		0,02		0,06	0,13
Intervalo de Confiança de 95%	0,94–1,00		0,94–1,00		0,85–1,00	0,61–1,00
Estatística z	42,1		42,1		16,7	6,5
Nível de Significância *p* (área = 0,5)	< 0,001		< 0,001		< 0,001	< 0,001
Valor de corte	12,3		12,0		14,2	
Especificidade	90%		90%		90,2%	81,0%
Sensibilidade	100%		100%		96,7%	80,4%
Exatidão total	95%		95%		93,4%	80,7%

* Procedimento de bootstrap realizado sem restituição. O modelo explicativo multivariado foi calculado no grupo Teste e validado no grupo Validação; ± = (média ± DP).

**Figura 2:**
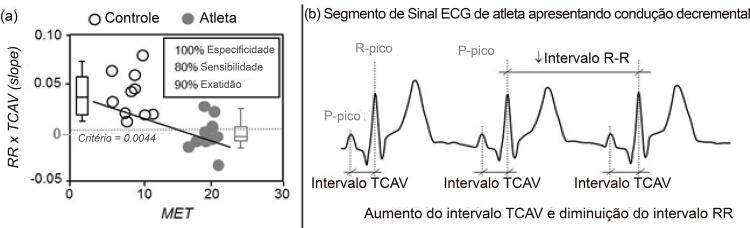
– *(a) Valores do slope da linha de regressão do TCAV combinado vs. intervalos RR médios (RR-TCAV_slope_) em função do consumo de VO2 expresso como equivalente metabólico máximo (MET) alcançado durante o teste de estresse e o respectivo gráfico boxplot. Observe que o RR-TCAV_slope_ tende a ser mais negativo à medida que o status do condicionamento físico aumenta (pontos cinzas), em comparação com indivíduos sedentários (pontos brancos). Gráficos boxplot mostrando mediana, intervalo interquartil e intervalos de confiança de 95% são mostrados nas proximidades dos respectivos pontos do grupo. Os valores de especificidade, sensibilidade e exatidão foram calculados utilizando-se o RR-TCAV_slope_ = 0,0044 como critério de corte. (b) Ilustração do segmento de ECG de atleta de 19 anos, representando uma sequência de batimentos sinusais normais, mostrando o alongamento do TCAV à medida que o intervalo RR diminui, indicando condução AV decremental. Observe os picos de onda P e onda R tomados como pontos fiduciais para avaliação do intervalo PR-pico. O TCAV foi avaliado como intervalo de PR-pico. TCAV - tempo de condução atrioventricular (veja o texto para maiores detalhes).*

Os valores de RR-TCAV_slope_ para cada grupo, incluindo faixa interquartil e intervalos de confiança (IC) de 95%, são mostrados na figura 2-a . A sensibilidade, a especificidade e a exatidão total foram calculadas utilizando-se o valor de corte ideal apresentado na tabela 1 e exibido como inserção. Para enfatizar a associação entre condução decremental espontânea e status físico, uma linha de regressão de RR-TCAV_slope_ vs. MET é apresentada na figura 2-a . Ela mostra correlação significativa (r = 0,70; p < 0,05) e slope negativo, demonstrando que o RR-TCAV_slope_ diminui à medida que o MET aumenta. Um exemplo de uma curta sequência de batimentos sinusais mostrando condução decremental espontânea, registrada durante o repouso em posição supina em um atleta de 19 anos (MET 16,8 METs) é mostrado na Figura 2-b .

## Discussão

A condução ventricular atrial é o determinante mais importante da duração do intervalo PR, que sofre flutuações dinâmicas, dependendo dos estados autonômicos e de saúde, idade, FC instantânea, medicamentos, postura e frequência respiratória.^17^ A avaliação do intervalo PR utilizando as abordagens de início de onda P de ou pico de onda P como pontos fiduciais demonstraram fornecer resultados concisos e precisos e, portanto, ambos são apropriados para avaliar as variações de TCAV inter-batimentos.^14 - 17^

No presente estudo, corredores de longa distância altamente treinados e indivíduos sedentários saudáveis tiveram sua potência aeróbica máxima avaliada e a variabilidade do TCAV acoplado ao intervalo RR anterior avaliada no ECG em repouso na posição supina. Foi levantada a hipótese de que, em repouso, a condução AV seria afetada pela remodelação AV induzida por treinamento avançado, fazendo com que o acoplamento do TCAV ao intervalo RR se comportasse de maneira diferente das condições sedentárias. Em um modelo linear, o acoplamento de TCAV ao intervalo RR mostrou um *slope* médio negativo da linha de regressão no grupo Atleta e, inversamente, um *slope* médio positivo no grupo Controle, indicando que a remodelação do nó AV devido ao treinamento induz aumento decrescente da condução. Uma medida potencialmente distinta da aptidão física, o *slope* de regressão do TCAV para o intervalo RR identificou corretamente 90% do status de aptidão física de todos os indivíduos. Embora a identificação de condução decremental em atletas seja um achado comum, que seja de nosso conhecimento, a aplicação de uma modelagem linear para quantificar TCAV e sua relação com o intervalo RR em atletas altamente treinados ainda não foi relatada.

Estudos anteriores demonstraram uma alta prevalência de bloqueio AV Mobitz I de 2º grau em corredores de longa distância em repouso.^8 - 10^ No presente estudo, a ocorrência de alongamento espontâneo do intervalo PR-pico relacionado ao encurtamento do intervalo RR foi um achado importante, tornando o *slope* médio negativo no grupo Atletas ( Figura 2b ). Ao contrário desses estudos, nenhuma onda P bloqueada foi encontrada após uma revisão cuidadosa dos sinais. Episódios espontâneos de condução decremental foram frequentes no grupo de Atletas (57,9% do tempo agregado de registro no ECG de Atletas) e raros no grupo Controle (7,9% do tempo agregado de registro no ECG dos Controles). Além disso, quando o slope de regressão de TCAV vs. intervalo RR foi plotado contra MET, observou-se que quanto maior o MET, mais negativo era o RR-TCAV_slope_, mostrando que a condução AV decremental espontânea se torna mais frequente à medida que o status do condicionamento físico melhora ( Figura 2a ) . Destaca-se que a condução decremental foi mais frequentemente observada quando o intervalo RR foi superior a 1.022,0 ms. A redução do intervalo PR-pico relacionada ao aumento do intervalo RR também foi observada nos atletas ( Figura 1d ). Uma possível explicação para esse último achado é a ocorrência comum de atividade de estimulação espontânea para-sinusal induzida vagalmente.

Foi demonstrado que o ECG de repouso de atletas de resistência pode mostrar características distintas de indivíduos sedentários saudáveis demograficamente equivalentes, tendo semelhanças com aquelas observadas em idosos e / ou pacientes com doença cardiovascular.^18^ Entretanto, em atletas, as anormalidades da condução AV têm sido relacionadas a maior atividade parassimpática, diferentemente dos idosos.^19^ Estudos atuais têm demonstrado que o treinamento atlético pode induzir adaptações fisiológicas intrínsecas no sistema de condução, contribuindo para a maior prevalência de anormalidades na condução AV.^11 - 13^ Os mecanismos fisiológicos pelos quais o treinamento de resistência induz essas alterações intrínsecas no sistema de condução cardíaca apresentam entendimento limitado e podem ser multifatoriais, mas alterações anatômicas, como dilatação atrial e ventricular, demonstraram a criação de um remodelamento mecânico-elétrico necessário para causar adaptações eletrofisiológicas AV intrínsecas.^7 , 11^

As limitações do presente estudo incluem o tamanho da amostra de dois grupos distintos em relação ao status de condicionamento físico. Os sinais brutos do ECG foram obtidos no banco de dados de ECG disponível no Programa de Engenharia Biomédica (amostra de conveniência). O intervalo Pico-P ao Pico-R foi utilizado como substituto da medida convencional do intervalo PR. Embora tenha sido demonstrado que o intervalo Pico-P ao Pico-R adequadamente descreve a dinamicidade do intervalo PR, a real duração do intervalo PR pode ser maior do que a observada no presente estudo. Observou-se que tanto a M-TCAV quanto o DP-TCAV foram maiores nos atletas quando comparados aos controles, embora a significância estatística não tenha sido alcançada. A explicação para esse achado pode ser dupla: i) embora se esperasse que a variabilidade do TCAV fosse maior entre os atletas, nenhum bloqueio Mobitz I verdadeiro foi observado após uma cuidadosa revisão do sinal. Isso indica que era esperado que a variabilidade do TCAV aumentasse até um certo limite e ii) o tamanho da amostra atual foi projetado para determinar diferenças relacionadas à energia total da ativação ventricular^6^ , limitando assim seu poder estatístico para detectar a variação do TCAV entre os grupos. Os indivíduos foram mantidos em repouso em posição supina por 10 minutos antes da aquisição do ECG, com o objetivo de impedir que o efeito da memória ortostática na condução AV influenciasse a condução AV na dinamicidade do acoplamento ao intervalo RR, em um ambiente com temperatura controlada e isolado acusticamente. No entanto, não se pode excluir completamente o fato de que algum efeito de memória ortostática ainda pudesse estar presente. Neste estudo, observou-se a ocorrência de aumento espontâneo do intervalo PR-pico à medida que o intervalo RR diminuiu e vice-versa. Esse fenômeno foi interpretado como uma manifestação de condução decremental durante o trânsito da frente de onda da ativação cardíaca através do nó AV. No entanto, devido à natureza deste estudo, nenhum teste eletrofisiológico invasivo foi realizado para melhor caracterizar a condução decremental ou a atividade de estimulação para-sinusal. Ainda é necessário investigar o potencial impacto dos achados atuais em contextos clínicos, como um marcador para taquiarritmias supraventriculares, particularmente a arritmia reentrante do nó AV e fibrilação atrial.

## Conclusão

O nó atrioventricular apresenta remodelação fisiológica substancial em corredores de longa distância de elite, caracterizada por condução AV decremental espontânea em repouso na posição supina, raramente observada em indivíduos sedentários saudáveis nas mesmas condições de repouso. O *slope* da linha de regressão linear do acoplamento PR-pico ao intervalo RR é uma variável explicativa forte e independente do equivalente metabólico máximo alcançado durante o teste de estresse nessa população.
