# Supplementary material for: Avaliação do Tempo de Condução Atrioventricular Dinâmica para Acoplamento ao Intervalo RR em Atletas e Indivíduos Sedentários
Source: Arq Bras Cardiol. 2020 Jul 28;115(1):71–7. [Article in Portuguese] doi: 10.36660/abc.20190281 (PMC8384324; doi:10.36660/abc.20190281)
Supplement: Translation

# Translation: Assessing Dynamic Atrioventricular Conduction Time to RR-interval Coupling in Athletes and Sedentary Subjects

**Authors:** Paulo Roberto Benchimol-Barbosa, Olivassé Nasario, Jurandir Nadal

**Affiliations:** 1 Brazil Hospital Universitário Pedro Ernesto, Coordenação Clínica, Rio de Janeiro, RJ – Brazil; 2 Brazil Universidade Federal do Rio de Janeiro, Instituto Alberto Luiz Coimbra de Pós-Graduação e Pesquisa de Engenharia, Rio de Janeiro, RJ – Brazil

**Keywords:** Athletes, Adults, Resistance Training, Physical Fitness, Ventricular Remodeling, Sedentarism, Electrocardiography/methods, Heart Ventricles, Ventricular Function

## Abstract

**Background:**

Atrioventricular conduction time ( *AVCT* ) is influenced by autonomic input and subject to physiological remodeling.

**Objective:**

To evaluate beat-by-beat *AVCT* and *RR-interval* variability in athletes and healthy sedentary subjects.

**Methods:**

Twenty adults, including 10 healthy sedentary (Controls) and 10 elite long-distance runners (Athletes), age, weight and height-adjusted, underwent maximal metabolic equivalent (MET) assessment, and 15-min supine resting ECG recording seven days later. The interval between P-wave and R-wave peaks defined the *AVCT* . Mean (M) and standard deviation (SD) of consecutive *RR-intervals (RR)* and coupled *AVCT* were calculated, as well as regression lines of RR vs. *AVCT (RR-AVCT)* . Concordant AV conduction was defined as positive *RR-AVCT slope* and discordant otherwise. A multivariate linear regression model was developed to explain MET based on *AVCT* and *RR-interval* variability parameters. Significance-level: 5 %.

**Results:**

In Athletes, *M-RR* and *SD-RR* values were higher than in Controls, whereas *M-AVCT* and *SD-AVCT* were not. *RR-AVCT* slopes were, respectively, 0.038 ± 0.022 and 0.0034 ± 0.017 (p < 0.05). Using a cut-off value of 0.0044 (AUC 0.92 ± 0.07; p < 0.001), *RR-AVCT* slope showed 100% specificity and 80% sensitivity. In a multivariate model, *SD-RR* and *RR-AVCT* slope were independent explanatory variables of MET (F-ratio: 17.2; p < 0.001), showing 100% specificity and 90% sensitivity (AUC 0.99 ± 0.02; p < 0.001).

**Conclusion:**

In elite runners, *AVCT* to *RR* -interval dynamic coupling shows spontaneous discordant *AV* conduction, characterized by negative *AVCT* vs. *RR* -interval regression line slope. *RR* -intervals standard deviation and *AVCT* vs. *RR* -interval regression line slope are independent explanatory variables of MET (Arq Bras Cardiol. 2020; [online].ahead print, PP.0-0)

## Introduction

Cardiac adaptation secondary to physical fitness is reflected in mechanical, electrical and autonomic remodeling of the heart, as a consequence of repeated high-demand activities. Well-trained athletes often have slight ventricular mass gain, increased ECG wave amplitude, early repolarization, reduction of resting heart rate and increased heart rate variability, related to the conditioning status.^1 -7^

Particularly, most autonomic heart remodeling in well-conditioned athletes is a consequence of increased vagal tonus and reduced sympathetic stimulation over the sinus and the atrioventricular (AV) nodes.^1 , 6^ Although increased vagal tonus may be straightforwardly detected by measuring the resting heart rate, to differentiate between increased parasympathetic activity over the sinus node and the AV node on surface ECG may not be that simple.

Frequently, high-demand athletes have atrioventricular (AV) node remodeling, characterized by several degrees of AV conduction block, non-sinus low atrial or junctional rhythm and, more rarely, complete AV block.^8-10^Those AV conduction disturbances depend on physical conditioning status and are related not only to increased parasympathetic activity over the AV node, but also to secondary remodeling of the AV node fibers and cell-to-cell coupling.^11-13^

Although *AV* conduction disturbances have been repeatedly documented in athletes, the dynamic *AV* conduction adaptation to the cardiac cycle in this population still needs clarification. In the general population, *AV* duration varies dynamically according to *RR-interval* duration, characterizing a concertina-like effect. However, in athletes, autonomic remodeling may influence dynamic *AV* conduction to *RR-interval* adaptation, leading to a distinct behavior of *AV* conduction, in a time-related response to *RR-interval* variation.

The present study evaluated beat-by-beat *AV* conduction time ( *AVCT* ) and *RR-interval* variabilities in elite runners and healthy sedentary subjects, at rest, aiming at assessing the effect of physical fitness status on spontaneous *AVCT* to *RR-interval* duration coupling.

## Methods

Detailed information about study protocol, Ethics Committee approval, and ECG data acquisition has been described elsewhere.^6^ The present study analyzed raw high resolution ECG data from the ECG data bank of the Biomedical Engineering Program, using a novel technique for extraction of atrioventricular conduction time and *RR-intervals* .^14^Data sampling procedure was described elsewhere.^6^

The study population comprised 20 volunteers divided in two groups: the ‘Athlete’ group, comprising ten elite long-distance runners (≥ 16.0 maximal metabolic equivalents [MET] calculated as the maximal oxygen consumption achieved during stress test divided by 3.5 ml·kg^-1^·min^-1^, [mean ± SD] 19.5 ± 1.3 MET; aged 25.1 ± 7.1 years), and the ‘Control’ group, comprising ten male healthy sedentary volunteers (≤ 11.5 MET; 8.7 ± 1.9 MET; aged 29.0 ± 5.4 years). It is worth mentioning that the term ‘MET’ is employed throughout the text as the maximal metabolic equivalent achieved during stress test. The athletes’ training program consisted of six to eight training sessions/week; 90 to 120 min/session; 90 to 110 km/week. The waves and fiducial point detection were carried out on ECG acquired using *XYZ* modified *Frank* leads, in the resting supine position, using low-pass filter at 15 Hz ( *Butterworth* , 4th order). For the analysis of the *RR-interval* duration, artefacts and ectopic beats were adequately excluded.^15,16^

The distance between the peak of the *P* -wave and the peak of the *R* -wave in normal beats defined the *PR-peak* interval and was employed to analyze instantaneous *AVCT* adaptation over the cardiac cycle.^14^The *PR-peak* to *RR-intervals* coupling was assessed in a beat-by-beat basis throughout the whole ECG recording, using the lead showing the tallest *P* -wave, usually the *Y* lead. The *RR-interval* was assessed as the time between the peaks of the R-waves of two consecutive normal beats. The *PR-peak* interval was assessed immediately before the second beat of the respective *RR-interval* . For each subject, the mean *(M)* and standard deviation *(SD)* of all consecutive normal *RR-intervals* ( *M-RR* and *SD-RR* ) and respective *PR-peak* interval ( *M-AVCT* and SD- *AVCT* ) were calculated. *PR-peak* intervals were correlated to the respective *RR-intervals* and calculated regression line slopes ( *RR-AVCT*_slope_).

### Statistical Analysis

The variables were expressed as mean ± SD or median and interquartile range, when appropriate. Data normality was assessed using Kolmogorov-Smirnov test, and all analyzed variables had their normality assumption accepted. Variables were compared between groups using non-paired Student’s t-test. To assess the optimal cut-off values, ROC curves were calculated from the regression line slopes ( *AVCT* vs. *RR-* interval) in both groups. A multivariate linear regression model was developed to explain the MET based on significant *AVCT* and *RR-interval* parameters. Pearson’s correlation coefficient r was tested for significance (significance level was set at 5%). A concordant AV conduction was arbitrarily defined as *AVCT* and *RR-interval* increased and decreased in the same direction in consecutive cardiac cycles, and discordant otherwise. *AVCT* was assessed as *PR-peak-interval* .

A validation bootstrap resampling procedure applied to the multivariate model was carried out using two different approaches. In the first approach, 1100 replications with replacement were carried out in the whole sample of both groups to assess mean and SD estimates of independent variables. In a second approach, both groups were split in a test group, comprising 67% of subjects of each group, and a validation group, with the remaining 33%. The MET estimated by the multivariate model was employed to classify Controls and Athletes in all sets of bootstrap procedures. Raw ECG signals were processed using custom-made programs written in Matlab R2007a (The MathWorks, Inc) language. Statistical analysis was carried out using MS-Excel 360 (Microsoft Corporation) and Medcalc version 11 (Medcalc Software bvba). The significance level adopted in the statistical analysis was 5%.

## Results

The Athletes had significantly higher *M-RR* and *SD-RR* values than the Controls, whereas there were no significant differences between *M-AVCT* and *SD-AVCT* values ( Table 1 ). Examples of subjects from the Control (a) and Athlete (b) groups are shown in Figure 1 , where regression lines and respective r of *AVCT* vs *RR-intervals* scatterplots are shown. *RR-AVCT*_slope_values are positive in Controls ( Figure 1-a ), whereas they are negative in Athletes ( Figure 1-b ). Overall, *RR-AVCT*_slope_in Controls and Athletes resulted in significant between-groups’ differences ( Table 1 ).

**Table 1:** – Univariate analyses of studied variables (mean ± SD)

	*M-RR*	*SD-RR*	*M-AVCT*	*SD-AVCT*	*RR-AVCT_slope_*
**Control group (mean ± SD)**	853.9 ± 94.0	44.5 ± 10.1	134.0 ± 17.7	2.8 ± 1.1	0.0376 ± 0.0219
**Athlete group (mean ± SD)**	1079.1 ± 207.9	76.4 ± 21.0	143.3 ± 27.6	3.8 ± 2.2	-0.0034 ± 0.0172
**p Significance level**	0.0084	0.0008	0.3820	0.2032	0.002
**ROC statistics**					
Area under the ROC curve (AUC)	0.89	0.9	0.56	0.61	0.92
Standard Error	0.07	0.08	0.14	0.14	0.06
95% Confidence Interval	0.67–0.98	0.68–0.98	0.32–0.78	0.37–0.82	0,71–0.99
z statistic	5.5	5.2	0.4	0.8	6.7
p Significance level (area=0.5)	< 0.0001	< 0.0001	0.6721	0.4177	< 0.0001
Cut-off value	917.3	60.9	124.1	3.8	0.0044
Specificity	80 %	100 %	40 %	50 %	100 %
Sensitivity	80 %	80 %	90 %	80 %	80 %
Accuracy	80 %	90 %	65 %	65 %	90 %

*M-RR: mean of all normal RR-intervals; SD-RR: standard deviation of all normal RR-intervals; M-AVCT: mean of PR-peak intervals respective to normal RR-intervals; SD-AVCT: standard deviation of PR-peak intervals respective to normal RR-intervals; RR-AVCTslope: slope of the linear regression line between PR-peak intervals and respective RR interval*

**Figure 1:** – *Scatterplot and regression line of beat-by-beat RR-interval as a function of respective PR-peak interval of a 30 y.o. Control subject (a) and a 19 y.o. Athlete (b). Two hundred heartbeat sequences of respective RR- and PR-peak intervals series are shown in (c) and (d). In (a), PR-peak interval increases as RR interval increases (positive slope: 0.0227; r = 0.50; p < 0.01), clearly observed in (c) (spontaneous concordant condition). Conversely, in (b), PR-peak interval decreases as RR interval increases (negative slope: -0.0316; r = -0.68; p < 0.01). In (d), note periods of reciprocal variation in RR- and PR-peak intervals (spontaneous discordant conduction): PR-peak interval shortens as RR-interval increases (dotted arrow) and PR-peak interval increases as RR-interval shortens (decremental conduction, solid arrow) (see text for details).*

Variables showing significant intergroup differences were entered into a multivariate linear regression model, taking MET as the dependent variable in the bootstrap procedure. *SD-RR* (p = 0.0099) and RR- *AVCT*_slope_(p = 0.006) were independent explanatory variables of MET, showing 90% specificity, 100% sensitivity and 95% total accuracy ( Table 2 ). The average C-statistic in test and validation groups were, respectively, 0.97 ± 0.06 and 0.87 ± 0.13; p < 0.001 for both. The multivariate linear regression analysis and respective bootstrap procedures results are summarized in Table 2 .

**Table 2:** – Multivariable explanatory model of the maximal VO2 consumption; 1100 bootstrap resampling procedure results and Internal validation of the maximal VO2 consumption multivariable explanatory model using bootstrap 2:1 ‘Test’ vs ‘Validation’ procedure results

	Multivariate model		Bootstrap procedure		Bootstrap test-validation	

Model Variables	Coefficients ±	*p*	Coefficients ±	*p*	Coefficients ±	*p*
**PR to RR slope (15 Hz)**	-100.36 ± 31.97	0.006	-105.76 ± 33.93	0.0009	-101.42 ± 29.39	0.0003
***SD RR***	0.115 ± 0.040	0.0099	0.115 ± 0.041	0.003	0.115 ± 0.036	0.0007
***Constant***	8.88		8.75 ± 3.22	0.003	8.85 ± 2.83	0.0009
ROC statistics					Test group	Validation group
Area under the ROC curve (AUC)	0.99		0.99		0.97	0.87
Standard Error	0.02		0.02		0.06	0.13
95% Confidence Interval	0.94–1.00		0.94–1.00		0.85–1.00	0.61–1.00
z statistic	42.1		42.1		16.7	6.5
*p* Significance level (area=0.5)	< 0.001		< 0.001		< 0.001	< 0.001
Cut-off value	12.3		12.0		14.2	
Specificity	90%		90%		90.2%	81.0%
Sensitivity	100%		100%		96.7%	80.4%
Total accuracy	95%		95%		93.4%	80.7%

* Procedimento de bootstrap realizado sem restituição. O modelo explicativo multivariado foi calculado no grupo Teste e validado no grupo Validação; ± = (média ± DP).

The RR- *AVCT*_slope_values for each group, including interquartile range and 95% confidence intervals (CI) are shown in figure 2-a . Sensitivity, specificity and total accuracy were computed utilizing the optimal cutoff value shown in table 1 , and exhibited as inset. To highlight the association between spontaneous decremental conduction and physical status, a regression line of RR- *AVCT*_slope_vs. MET is shown in figure 2-a . It shows a significant correlation (r = 0.70; p < 0.05) and a negative slope, demonstrating that RR- *AVCT*_slope_decreases as MET increases. An example of a short sequence of sinus beats showing spontaneous decremental conduction, registered during supine rest in a 19 y.o. athlete (MET 16.8 METs) is shown in Figure 2-b .

**Figure 2:** – *(a) Regression line slope values of pooled AVCT vs. mean RR-intervals (RR-AVCTslope) as a function of VO2 consumption expressed as maximal metabolic equivalent (MET) achieved during stress test and respective boxplot. Note that RR-AVCTslope tends to be more negative as physical conditioning status increases (grey dots), when compared to sedentary individuals (white dots). Box-plots showing median, interquartile range and 95% confidence intervals are shown in the vicinity of the respective group points. Specificity, sensitivity and accuracy values were computed utilizing RR-AVCTslope = 0.0044 as a cut-off criterion. (b) Illustration of 19 y.o. Athlete’s ECG segment depicting a sequence of normal sinus beats showing AVCT lengthening as RR-interval decreases, indicating AV decremental conduction. Note P-wave and R wave peaks taken as the fiducial points for assessment of the PR-peak interval. AVCT was assessed as PR peak interval. AVCT – atrioventricular conduction time (see text for details).*

## Discussion

Atrial ventricular conduction is the most important determinant of the *PR-interval* duration, which undergoes dynamic fluctuations depending on autonomic and health statuses, age, instantaneous HR, medications, stance and respiratory frequency.^17^The evaluation of the *PR-interval* by using either *P* wave-onset or *P* wave-peak approaches as fiducial points has been shown to provide accurate and precise results, and, thus, are both appropriate to assess *AVCT* inter-beat variations.^14,17^

In the present study, highly trained longdistance runners and healthy sedentary subjects had their maximal aerobic power assessed, and *AVCT* coupled to the preceding *RR-interval* variability, assessed on resting supine ECG. It was hypothesized that, at rest, AV conduction would be affected by AV remodeling induced by high-end training, causing *AVCT* to *RR-interval* coupling to behave differently from sedentary conditions. In a linear model, *AVCT* to *RR-interval* coupling showed an average negative regression line slope in the Athlete group and, conversely, an average positive slope in the Control group, indicating that AV node remodeling due to training induces decremental conduction enhancement. A potentially distinguished measure of physical fitness, *AVCT* to *RR-interval* regression slope correctly identified 90% all subjects’ related physical fitness status. Although identification of decremental conduction in athletes is a common finding, the application of a linear modeling to quantify *AVCT* and its relation to *RR-interval* in highly trained athletes has not been yet reported, to the best of our knowledge.

Previous studies evidenced a high prevalence of Mobitz I 2nd degree AV block in long distance runners at rest.^8-10^In the present study, the occurrence of spontaneous *PR-peak-interval* lengthening related to *RR-interval* shortening was a major finding, making the average slope negative in the Athletes group ( Figure 2b ). Conversely to these studies, no blocked *P* -wave was found after a carefully revision of signals. Spontaneous episodes of decremental conduction were frequent in the Athletes group (57.9% of aggregated ECG recording time of Athletes) and rare in the Control group (7.9% of aggregated ECG time recording of Controls). Furthermore, when the *AVCT* vs. *RR-interval* regression slope was plotted against MET, it was observed that the higher the MET, the more negative the RR- *AVCT*_slope_, showing that spontaneous decremental AV conduction becomes more frequent as physical conditioning status improves ( Figure 2a ). Noteworthy was that the decremental conduction was more frequently observed when *RR-interval* was larger than 1022.0 ms. *PR-peak* interval reduction related to RR interval increase was also observed in Athletes ( Figure 1d ). A possible explanation for this latter finding is the common occurrence of vagal-induced spontaneous para-sinus pacing activity.

It has been shown that the resting ECG of endurance athletes may show distinctive features from demographically equivalent healthy sedentary subjects, bearing similarities to those observed in elderly individuals and/or patients with cardiovascular disease.^18^However, in athletes, AV conduction abnormalities have been related to higher parasympathetic activity, differently from the elderly.19 Contemporary studies have shown that athletic training could induce intrinsic physiological adaptations in the conduction system, contributing to the higher prevalence of AV conduction abnormalities.^11-13^The physiological mechanisms by which endurance training induces those intrinsic changes in the cardiac conduction system are limitedly understood and may be multifactorial, but anatomic changes such as atrial and ventricular dilation has been shown to create a mechanical-to-electrical remodeling necessary to cause intrinsic AV electrophysiological adaptations.^7,11^

Limitations of the present study include the sample size of two distinct groups regarding the physical conditioning status. Raw ECG signals were obtained from the ECG database available in the Biomedical Engineering Program (convenience sample). *Peak-P* to *Peak-R* interval was employed as a surrogate of conventional *PR-interval* measurement. Although it has been shown that *Peak-P* to *Peak-R* interval appropriately describes *PR-interval* dynamicity, the duration of the actual *PR-interval* may be larger than the one observed in the present study. It was observed that both *M-AVCT* and *SD-AVCT* were larger among athletes when compared to controls, although statistical significance was not reached. The explanation of this finding may be twofold: i) although *AVCT* variability was expected to be higher among athletes, no true Mobitz I block was in fact observed after careful signal revision. This indicates that *AVCT* variability was expected to be increased to a limited extent, and ii) the sample size of the present sample was designed to determine differences related to ventricular activation total energy^6^ , thus limiting its statistical power to detect *AVCT* variation between groups. Subjects were kept on supine rest for 10 minutes before ECG acquisition, aiming at preventing orthostatic memory effect on AV conduction to influence AV conduction to *RR-interval* coupling dynamicity, in a controlled temperature and acoustically isolated environment. However, it cannot be completely ruled out that some orthostatic memory effect might still be present. In this study, we observed the occurrence of spontaneous *PR-peak* interval enlargement as *RR-interval* decreased, and vice-versa. This phenomenon was interpreted as a manifestation of decremental conduction during the transit of the cardiac activation wave-front through the AV node. However, due to the nature of this study, no invasive electrophysiological test was carried out to further characterize decremental conduction or para-sinus pacing activity. It is still necessary to investigate the potential impact of the present findings on clinical settings, such as a marker for supraventricular tachyarrhythmia, particularly AV nodal reentrant arrhythmia and atrial fibrillation.

## Conclusion

The atrioventricular node undergoes substantial physiological remodeling in elite long-distance runners, characterized by spontaneous AV decremental conduction at supine rest, rarely observed in healthy sedentary subjects under the same resting conditions. The linear regression line slope of *PR-peak* to *RR-interval* coupling is a strong and independent explanatory variable of maximal metabolic equivalent achieved during stress test in this population.
